# Supplementary material for: Short communication: Timber harvesting impacts small mammal foraging behavior and larval tick infestation
Source: PLoS One. 2025 Jun 3;20(6):e0325495. doi: 10.1371/journal.pone.0325495 (PMC12133162; doi:10.1371/journal.pone.0325495)
Supplement: Attachment — Submitted filename: Review_PLOS_PONE D 24 45269.pdf [file pone.0325495.s001.pdf]

Review: PONE-D-24-54269, short communication

Hurd and Gardner. Timber harvesting impacts small mammal foraging behavior and tick infestation.

Although inclined to accept this manuscript with revisions, there are edits and questions that should be addressed by the authors.

General comments

One question that occurs to me is why this study, collected during one month in 2021, merits a stand alone manuscript, when this is obviously part of a larger study that was recently published and cited in the text (Hurd et al 2024, J Med Ent). Based on the cited study, the overarching project was a two-year endeavor, is there not mammal data for this entire project period?

The authors include the species commonly know as the boreal red-backed vole in this ms. Note that the scientific name has been officially changed back to Clethrionomys from Myodes in 2019. See Krystufek, B. (2019). "Back to the future: the proper name for red-backed voles is Clethrionomys Tilesius and not Myodes Pallas". Mammalia. 84 (2): 214–217. doi:10.1515/mammalia-2019-0067

Given that the manuscript is derived from data in August, are most of the data for ticks dealing with larvae? Or is there data on nymphs as well? If yes, they should be separated. If there is not data to examine nymphs, then the authors should make this clear in the text and may want to include 'larval tick infestation' in the title.

I would suggest a more detailed description of the type of harvests examined, and to consistently refer to them directly in the ms. As an example, discussing forage trays 'covered by understory vegetation' versus, presumably, high intensity harvest stands (lines 105-107)?

Broadly, this manuscript is about forest management techniques, but there is no information on the forest type or species composition, both of which may play some role in small mammal activity. For instance, the seasonal effect of seed production in a stand may be different if the producer is an American beech (Fragus grandifolia) versus a northern red oak (Quercus rubra). As this study was only conducted over a short period, for one month, the forest composition is a vital fact that has been left out. Similarly, where was this study conducted? Forest plots located on the southern coast may be very different from what are seen in northern industrial forests. Such factors may also influence the establishment and population size of Ix. scapularis, which seems to still be expanding its range in northern New England states. Based on the initial study cited in the text (Hurd et al. 2024), research occurred across, at least, three counties in Maine, some of which are geographically disparate.

Although this study focuses on foraging behavior, any information on nesting behavior, which may also play a role in co-feeding, as well as maintenance of the host-pathogen cycle?

Finally, given that one of the key factors in limitation or mortality in Ix. scapularis is desiccation, is there micro-humidity data present for the study grids between the different forest harvests? It would seem that this is an important question, if the investigators were looking at encounters

between hosts and questing ticks. Since the authors cites the use of dataloggers in the original manuscript, cited above, why was that data not included here?

Specifically, edits to be considered:

Line 57: Should the term reservoir host be used?

Methods section, line 67-95: It is stated later that camera traps were used, this should be included in this section, as should a description of the feeding trays. The authors do state later in the ms that the question of what types of animals visited the feeders are addressed by the use of cameras. They may want to include this earlier as it could be an important confounder if birds or larger mammals, such as raccoons were also visiting feeders.

Results section, lines 114-122: What was the distance between sampling grids? Any issue with migration from one area to another. That may address the uniformity of infection across the study.

No data on the chipmunks???

Discussion section, lines 144-146: Maybe be specific about the species of squirrels discussed. Are you including chipmunks, which is a type of sciurid? Gray and red squirrels may differ greatly in their tick burdens as well, as one is more arboreal than the other.
