# Supplementary material for: Short communication: Timber harvesting impacts small mammal foraging behavior and larval tick infestation
Source: PLoS One. 2025 Jun 3;20(6):e0325495. doi: 10.1371/journal.pone.0325495 (PMC12133162; doi:10.1371/journal.pone.0325495)
Supplement: Attachment — Submitted filename: Response To Reviewers.docx [file pone.0325495.s002.docx]

To the Editor:

We appreciate the response provided by *PLOS One* and the opportunity to revise and resubmit our manuscript and incorporate the thoughtful feedback provided by yourself and the reviewer. Below we provide in-depth responses to the concerns raised and indicate changes made to our manuscript in response to these comments. The line numbers mentioned in our replies to reviewer comments refer to the lines in the document containing tracked changes.

In response to the primary concerns, we have 1) explained the choice to include only larval ticks in our analyses, 2) discussed deer habitat use, 3) described the distinction between this current study and our previous one, and 4) elaborated on our methods and study site descriptions.

Thank you for your consideration and we look forward to your feedback.

Sincerely,

Stephanie Hurd and Allison Gardner

**Response to Editor Comments**

Journal requirements:

When submitting your revision, we need you to address these additional requirements.

1. Please ensure that your manuscript meets PLOS ONE's style requirements, including those for file naming. The PLOS ONE style templates can be found at

https://journals.plos.org/plosone/s/file?id=wjVg/PLOSOne_formatting_sample_main_body.pdf and

https://journals.plos.org/plosone/s/file?id=ba62/PLOSOne_formatting_sample_title_authors_affiliations.pdf

>>>We have made the necessary changes to ensure our manuscript meets all style requirements, including revising the title page, formatting the entire document, fixing our references, and renaming files.

2. Thank you for stating the following in the Acknowledgments Section of your manuscript:

“We thank B. Stevens for his assistance with field data collection, the University of Maine Cooperative Extension Tick Lab for performing tick-borne pathogen detection in small mammal tissue samples, and members of the Gardner Lab at the University of Maine for their helpful feedback and support. We thank the U.S. Forest Service Northern Research Station, Maine Woodland Owners, Portland Water District, and individual property owners for land use permission. This study was supported by a USDA National Institute of Food and Agriculture grant (Project No. ME012450318) to AMG and Hatch award through the Maine Agricultural and Forest Experiment Station (Project No. ME032025) to AMG.”

We note that you have provided funding information that is currently declared in your Funding Statement. However, funding information should not appear in the Acknowledgments section or other areas of your manuscript. We will only publish funding information present in the Funding Statement section of the online submission form.

Please remove any funding-related text from the manuscript and let us know how you would like to update your Funding Statement. Currently, your Funding Statement reads as follows:

“This study was supported by a USDA National Institute of Food and Agriculture grant (Project No. ME012450318; https://www.nifa.usda.gov/) to AMG and Hatch award through the Maine Agricultural and Forest Experiment Station (Project No. ME032025; https://umaine.edu/mafes/) to AMG. No funders played a role in the study design, data collection or analysis, decision to publish, or manuscript preparation.”

Please include your amended statements within your cover letter; we will change the online submission form on your behalf.

>>>We have revised our Acknowledgments section to remove the function information (Lines 296-302). The Funding Statement above contains all necessary information and does not need to be updated.

3. Please include your full ethics statement in the ‘Methods’ section of your manuscript file. In your statement, please include the full name of the IRB or ethics committee who approved or waived your study, as well as whether or not you obtained informed written or verbal consent. If consent was waived for your study, please include this information in your statement as well.

>>>We have included our full ethics statement in the Methods section (Lines 160-162).

Additional Editor Comments:

I agree with the reviewer's comments; although only one reviewer responded, in the interest of providing you with a timely review I have read the ms myself. I completely agree with the reviewer that the developmental stage of the tick needs to be specified. Indeed, if the trapping was done in August, it is likely that the ticks that are the focus of the analysis are larval deer ticks. This begs the question as to whether differential habitat use by deer has influenced the distribution and abundance of ticks across the two sites. Deer would drop engorged female ticks where they browse the most; such ticks would produce larvae that would infest small mammals in the site. If the ticks infesting small mammals were nymphs, they might have been acquired as larvae elsewhere and transported across sites to drop and develop into nymphs. There needs to be discussion of the potential confounding role of deer in explaining the results that are reported. There also needs to be discussion as to why previously published data requires another analysis and publication.

>>>Thank you for this comment. All the focus of the analysis were larval ticks. We agree it is necessary to make this clear and have clarified the results (Lines 209-210) and changed the manuscript title.

We also agree it is important to address the role of deer in this system, and explain deer are an unlikely confounding variable as past work showed deer habitat use was constant across all sites (Lines 133-133).

While this study uses the same experimental units referred to in our previous publication, the small mammal work was a separate study conducted on only a subset of the total original experimental units (n = 14) after the completion of the prior study in 2020. We attempt to make clear this separation and clarify that we reference the previous study to help speculate on the underlying mechanisms that may explain the findings of this separate project (Lines 100-102).

**Response to Reviewer Comments**

General comments

One question that occurs to me is why this study, collected during one month in 2021, merits a

stand alone manuscript, when this is obviously part of a larger study that was recently published

and cited in the text (Hurd et al 2024, J Med Ent). Based on the cited study, the overarching project was a two-year endeavor, is there not mammal data for this entire project period?

>>>Thank you for this comment. No, there is no mammal data for the project that occurred from 2019 – 2020. This small mammal study during 2021 addressed here occurred after the completion of the prior project in 2020. The past study (Hurd et al 2024, J Med Ent) focused on forest structural conditions that result from timber harvesting, microclimate, and the role of other hosts (i.e., deer), but did not address the role of small mammals. Therefore, we felt this gap warranted an additional study to examine how timber harvesting may affect small mammal communities, and, in turn, be able to speculate if these effects could have consequences for the differences in tick populations that we discovered in the prior study (Lines 100-102).

The authors include the species commonly know as the boreal red-backed vole in this ms. Note

that the scientific name has been officially changed back to Clethrionomys from Myodes in 2019. See Krystufek, B. (2019). "Back to the future: the proper name for red-backed voles is

Clethrionomys Tilesius and not Myodes Pallas". Mammalia. 84 (2): 214–217.

doi:10.1515/mammalia-2019-0067

>>>Thank you for catching this error! We have corrected the scientific name (Line 205).

Given that the manuscript is derived from data in August, are most of the data for ticks dealing with larvae? Or is there data on nymphs as well? If yes, they should be separated. If there is not data to examine nymphs, then the authors should make this clear in the text and may want to include ‘larval tick infestation’ in the title.

>>>Thank you for this feedback. Yes, all the tick data deals only with larvae as there was very limited detection of nymphs or adults. We agree it is necessary to make this clear and have clarified the results (Lines 209-210) and changed the title.

I would suggest a more detailed description of the type of harvests examined, and to consistently

refer to them directly in the ms. As an example, discussing forage trays ‘covered by understory

vegetation’ versus, presumably, high intensity harvest stands (lines 105-107)?

>>>Thank you for this comment. Within each harvest intensity treatment (low vs. high) we deployed foraging trays that were either covered or uncovered by understory vegetation, for a total of four distinct conditions: (1) low harvest/covered, (2) low harvest/uncovered, (3) high harvest/covered, and (4) high harvest/uncovered. We have added a description of this design to the methods section to improve clarity (Lines 169-174).

Broadly, this manuscript is about forest management techniques, but there is no information on the forest type or species composition, both of which may play some role in small mammal activity. For instance, the seasonal effect of seed production in a stand may be different if the producer is an American beech (Fragus grandifolia) versus a northern red oak (Quercus rubra). As this study was only conducted over a short period, for one month, the forest composition is a vital fact that has been left out. Similarly, where was this study conducted? Forest plots located on the southern coast may be very different from what are seen in northern industrial forests. Such factors may also influence the establishment and population size of Ix. scapularis, which seems to still be expanding its range in northern New England states. Based on the initial study cited in the text (Hurd et al. 2024), research occurred across, at least, three counties in Maine, some of which are geographically disparate.

>>>Thank you for this review, we agree additional study site description is needed. We have added additional information to the methods section about study location, forest type, and tree species composition (Lines 128-136).

Although this study focuses on foraging behavior, any information on nesting behavior, which may also play a role in co-feeding, as well as maintenance of the host-pathogen cycle?

>>>Thank you for this point, nesting behavior could also be an interesting component of this system. We did not gather any data on nesting behavior during this study, but agree it warrants consideration and have addressed it in the discussion section (Lines 273-279).

Finally, given that one of the key factors in limitation or mortality in Ix. scapularis is desiccation, is there micro-humidity data present for the study grids between the different forest harvests? It

would seem that this is an important question, if the investigators were looking at encounters

between hosts and questing ticks. Since the authors cites the use of dataloggers in the original

manuscript, cited above, why was that data not included here?

>>>Thank you for this comment, we have cited our microclimate data findings from our previous study (Lines 284-286).

Specifically, edits to be considered:

Line 57: Should the term reservoir host be used?

>>>We believe the term ‘host’ here is appropriate rather than ‘reservoir host’ because in this sentence we discuss small mammals as hosts for ectoparasites rather than as reservoir hosts for tickborne pathogens like *Borrelia burgdorferi*. The term reservoir host refers to the incubation and persistence of the pathogen, not the vector.

Methods section, line 67-95: It is stated later that camera traps were used, this should be included in this section, as should a description of the feeding trays. The authors do state later in the ms that the question of what types of animals visited the feeders are addressed by the use of cameras. They may want to include this earlier as it could be an important confounder if birds or larger mammals, such as raccoons were also visiting feeders.

>>>Thank you for this suggestion, we agree more detailed methods are necessary. We have added descriptions of the trays with trail cameras as well as the track plates (Lines 169-198). From the trail camera photos of the foraging trays, the only animals removing seed from the trays appeared to be small mammals like mice and possibly voles or shrews. There were two brief instances of raccoons inspecting the foraging trays, but the captured photos indicate no seed, or very limited seed, was removed during these times and the encounters were purely investigatory.

Results section, lines 114-122: What was the distance between sampling grids? Any issue with

migration from one area to another. That may address the uniformity of infection across the study.

>>>Thank you for this comment. We have added the distance between sampling grids (500 m) to the methods section (Line 130-131).

No data on the chipmunks???

>>>We chose to restrict our analyses to just *Peromyscus leucopus* data as chipmunk captures were so rare (only 10 captures study-wide, or less than 4% of total captures) and we only managed to gather full observations (i.e., tick burdens, ear tags, and ear biopsies) from 4 out of the 10 chipmunks. We felt the data was not substantial enough for robust analyses. We have added an explanation for the lack of chipmunk data to the results section (Lines 207-209).

Discussion section, lines 144-146: Maybe be specific about the species of squirrels discussed. Are you including chipmunks, which is a type of sciurid? Gray and red squirrels may differ greatly in their tick burdens as well, as one is more arboreal than the other.

>>>This is an important point, and we agree that specificity is required. We have included this detail to further clarify the findings from the studies we cited (Line 270).
